# The Generation R Study: design and cohort update 2017

**DOI:** 10.1007/s10654-016-0224-9

**Published:** 2017-01-09

**Authors:** Marjolein N. Kooijman, Claudia J. Kruithof, Cornelia M. van Duijn, Liesbeth Duijts, Oscar H. Franco, Marinus H. van IJzendoorn, Johan C. de Jongste, Caroline C. W. Klaver, Aad van der Lugt, Johan P. Mackenbach, Henriëtte A. Moll, Robin P. Peeters, Hein Raat, Edmond H. H. M. Rings, Fernando Rivadeneira, Marc P. van der Schroeff, Eric A. P. Steegers, Henning Tiemeier, André G. Uitterlinden, Frank C. Verhulst, Eppo Wolvius, Janine F. Felix, Vincent W. V. Jaddoe

**Affiliations:** 1 The Generation R Study Group (NA-2915), Erasmus Medical Center, University Medical Center, PO Box 2040, 3000 CA Rotterdam, The Netherlands; 2 Department of Epidemiology, Erasmus Medical Center, University Medical Center, PO Box 2040, 3000 CA Rotterdam, The Netherlands; 3 Department of Pediatrics, Erasmus Medical Center, University Medical Center, PO Box 2040, 3000 CA Rotterdam, The Netherlands; 4 Division of Respiratory Medicine and Allergology, Department of Pediatrics, Erasmus Medical Center, University Medical Center, PO Box 2040, 3000 CA Rotterdam, The Netherlands; 5 Division of Neonatology, Department of Pediatrics, Erasmus Medical Center, University Medical Center, PO Box 2040, 3000 CA Rotterdam, The Netherlands; 6 Center for Child and Family Studies, Leiden University, Leiden, The Netherlands; 7 Department of Psychology, Education and Child Studies, Erasmus University Rotterdam, Rotterdam, The Netherlands; 8 Department of Ophthalmology, Erasmus Medical Center, University Medical Center, PO Box 2040, 3000 CA Rotterdam, The Netherlands; 9 Department of Radiology, Erasmus Medical Center, University Medical Center, PO Box 2040, 3000 CA Rotterdam, The Netherlands; 10 Department of Public Health, Erasmus Medical Center, University Medical Center, PO Box 2040, 3000 CA Rotterdam, The Netherlands; 11 Department of Internal Medicine, Erasmus Medical Center, University Medical Center, PO Box 2040, 3000 CA Rotterdam, The Netherlands; 12 Department of Otolaryngology, Head and Neck Surgery, Erasmus Medical Center, University Medical Center, PO Box 2040, 3000 CA Rotterdam, The Netherlands; 13 Department of Obstetrics and Gynecology, Erasmus Medical Center, University Medical Center, PO Box 2040, 3000 CA Rotterdam, The Netherlands; 14 Department of Child and Adolescent Psychiatry, Erasmus Medical Center, University Medical Center, PO Box 2040, 3000 CA Rotterdam, The Netherlands; 15 Department of Oral and Maxillofacial Surgery, Special Dental Care and Orthodontics, Erasmus Medical Center, University Medical Center, PO Box 2040, 3000 CA Rotterdam, The Netherlands

**Keywords:** Cohort study, Epidemiology, Pregnancy, Child, Adolescence

## Abstract

The Generation R Study is a population-based prospective cohort study from fetal life until adulthood. The study is designed to identify early environmental and genetic causes and causal pathways leading to normal and abnormal growth, development and health from fetal life, childhood and young adulthood. This multidisciplinary study focuses on several health outcomes including behaviour and cognition, body composition, eye development, growth, hearing, heart and vascular development, infectious disease and immunity, oral health and facial growth, respiratory health, allergy and skin disorders of children and their parents. Main exposures of interest include environmental, endocrine, genomic (genetic, epigenetic, microbiome), lifestyle related, nutritional and socio-demographic determinants. In total, 9778 mothers with a delivery date from April 2002 until January 2006 were enrolled in the study. Response at baseline was 61%, and general follow-up rates until the age of 10 years were around 80%. Data collection in children and their parents includes questionnaires, interviews, detailed physical and ultrasound examinations, behavioural observations, lung function, Magnetic Resonance Imaging and biological sampling. Genome and epigenome wide association screens are available. Eventually, results from the Generation R Study contribute to the development of strategies for optimizing health and healthcare for pregnant women and children.

## Introduction

The Generation R Study is a population-based prospective cohort study from fetal life until young adulthood. The background and design have been described in detail previously [1–7]. Briefly, the Generation R Study is designed to identify early environmental and genetic causes of normal and abnormal growth, development and health from fetal life until young adulthood. This multidisciplinary study focuses on several health outcomes including behaviour and cognition, body composition, eye development, growth, hearing, heart and vascular development, infectious disease and immunity, oral health and facial growth, respiratory health, allergy and skin disorders of children and their parents. Main exposures of interest include environmental, endocrine, genomic (genetic, epigenetic, microbiome) lifestyle related, nutritional and socio-demographic determinants. Full lists of exposures and outcomes are presented in Tables 1 and 2. An important focus of the study is on the identification of new early life determinants of common non-communicable diseases in adulthood or there risk factors, on which various papers have been published recently in this journal [8–26]. A detailed and extensive data collection has been conducted over the years, starting in the early prenatal phase and currently in early adolescence (age 13 years). Data collection in parents and their children included questionnaires, interviews, detailed physical and ultrasound examinations, behavioural observations, lung function, Magnetic Resonance Imaging (MRI) and biological sampling. In this paper, we give an update of the data collection in the children and their parents until the child’s age of 13 years.

**Table 1:** Main outcomes per research area

Maternal health	Cardiovascular health
	Endothelial (dys)function
	Pregnancy complications
	Risk factors for osteoporosis
	Risk factors for type 2 diabetes
Growth and physical development	Body composition and obesity
	Bone development
	Childhood growth patterns
	Dental development
	Dental caries
	Fetal growth patterns and organ development
	Myopia
	Physical characteristics and appearance
	Puberty stages
	Risk factors for cardiovascular disease
	Risk factors for type 2 diabetes
Behavioural and cognitive development	Attachment
	Behavioural and emotional problems
	Brain development
	Child psychopathology
	Child risk taking behaviour (alcohol, drugs, smoking)
	Child physical activity and sedentary behaviours
	Child sleeping patterns
	Compliance and moral development
	Family interaction, parenting and child attachment
	Language delay
	Neuromotor development
	Neuropsychology—executive function
	Stress reactivity
	Use of social media
	Verbal and nonverbal cognitive development
Airways, asthma, allergy and skin disordes	Airways and lung structure
	Acne
	Allergy
	Asthma
	Eczema
	Hearing loss
	Lung function
	Physical (exercise) condition
	Microbiome skin
	Skin color
Infectious and inflammatory diseases	Celiac disease
	Infectious diseases and immune system
Health and healthcare	Health care utilization
	Social health inequalities
	Qualitiy of life

**Table 2:** Main determinants

Endocrine determinants	Maternal and fetal thyroid hormone levels
	Maternal thyroid autoimmunity
	Maternal hCG levels
	Childhood thyroid hormone and cortisol levels
Environmental determinants	Air pollution during pregnancy and childhood (PM10, NO_2_)
	Bisphenol A, pesticides, phthalates
	Housing conditions
	Home environment
Genetic, epigenetic and microbiome determinants	Genetic variants (genome wide, candidate gene) DNA methylation (genome wide, candidate gene)
Lifestyle related determinants	Parental alcohol consumption
	Parental anthropometrics and obesity
	Parental smoking
	Parental working conditions
	Child anthropometrics and obesity
	Child music listening behaviour
	Child sedentary and physical activity behaviour
	Child smoking
	Dental care
Nutritional determinants	Maternal nutrition (products, patterns)
	Folic acid supplement use
	Breastfeeding
	Infant and childhood nutrition (timing, products, patterns)
	Nutritional biomarkers (folate, homocystein, vitamin B12, vitamin D)
Infection and micriobiota	Nasopharyngeal microbiota and bacterial carriage
	Faeces microbiota
Social-demographic determinants	Ethnicity
	Parental education, employment status and household income
	Parental marital status
	Parental psychopathology

## Study design

The Generation R Study is conducted in Rotterdam, the second largest city in the Netherlands. Rotterdam is situated in the Western part of the Netherlands. The study is a population-based prospective cohort study from fetal life onwards. Pregnant women with an expected delivery date between April 2002 and January 2006 living in Rotterdam were eligible for participation in the study. Extensive assessments are performed in mothers, fathers and their children. Measurements were planned in early pregnancy (gestational age <18 weeks), mid pregnancy (gestational age 18–25 weeks) and late pregnancy (gestational age >25 weeks). The fathers were assessed once during the pregnancy of their partner. The children form a prenatally recruited birth cohort that will be followed at least until young adulthood. In the preschool period, which in the Netherlands refers to the period from birth until the age of 4 years, data collection was performed by a home-visit at the age of 3 months, and by repeated questionnaires and routine child health centers visits. Information from these routine visits was obtained and used for the study. Additional detailed measurements of fetal and postnatal growth and development were conducted in a randomly selected subgroup of Dutch children and their parents at a gestational age of 32 weeks and postnatally at the ages of 1.5, 6, 14, 24, 36 and 48 months in a dedicated research center.

Around the ages of 6 and 10 years all children and their parents were invited to visit our research center in the Erasmus MC-Sophia Children’s Hospital to participate in hands-on measurements, advanced imaging modalities, behavioural observations and biological sample collection. MRI scans of all participating children were made in order to image abdominal composition, brain, lungs, cardiovascular system, fat tissue, kidney, liver, and hip development. Furthermore, the parents received 6 questionnaires during this period. Children also received their own questionnaire around the age of 10. Information from municipal health services, schools and general practicionars has also been collected.

In the current adolescence period, all children and their parents will be re-invited around the child’s age of 13 and 16 years. We will again assess their growth, development and health in our research center and with questionnaires. We will perform MRI scans of the abdominal composition (fat), brain, and hip development.

## Study cohort

### Eligibility and enrolment

Eligible mothers were those who were resident in the study area at their delivery date and had an expected delivery date from April 2002 until January 2006. We aimed to enrol mothers in early pregnancy but enrolment was possible until birth of their child. The enrolment procedure has been described previously in detail [1–4]. In total, 9778 mothers were enrolled in the study. Of these mothers, 91% (n = 8879) was enrolled during pregnancy. Partners from mothers enrolled in pregnancy were invited to participate. In total, 71% (n = 6347) of all fathers were included. A total of 1232 pregnant women and their children form the subgroup of Dutch children for additional detailed studies. The overall response rate based on the number of children at birth was 61%.

The study group is an multi ethnic cohort. Ethnicity was defined according the classification of Statistics Netherlands [27–32]. Ethnic background was assessed in accordance with the country of birth of participants themselves and his or her parents. A participant was considered to have non-Dutch ethnic origin if one of her parents was born abroad. If both parents were born abroad, the country of birth of the participant’s mother determined the ethnic background [33]. The largest ethnic groups were the Dutch, Surinamese, Turkish and Moroccan groups. We also constructed a dichotomous variable “Western/non-Western”ethnicity. Western ethnicity included Dutch, European, American Western (including North American), Asian Western (including Indonesian and Japanese) and Oceanian. Non-Western ethnicity included Turkish, Moroccan, Surinamese, Antillean, Cape Verdean, African, Asian (except Indonesia and Japan) and South American and Central American [33, 34].

### Response and follow-up

**Fig. 1:**
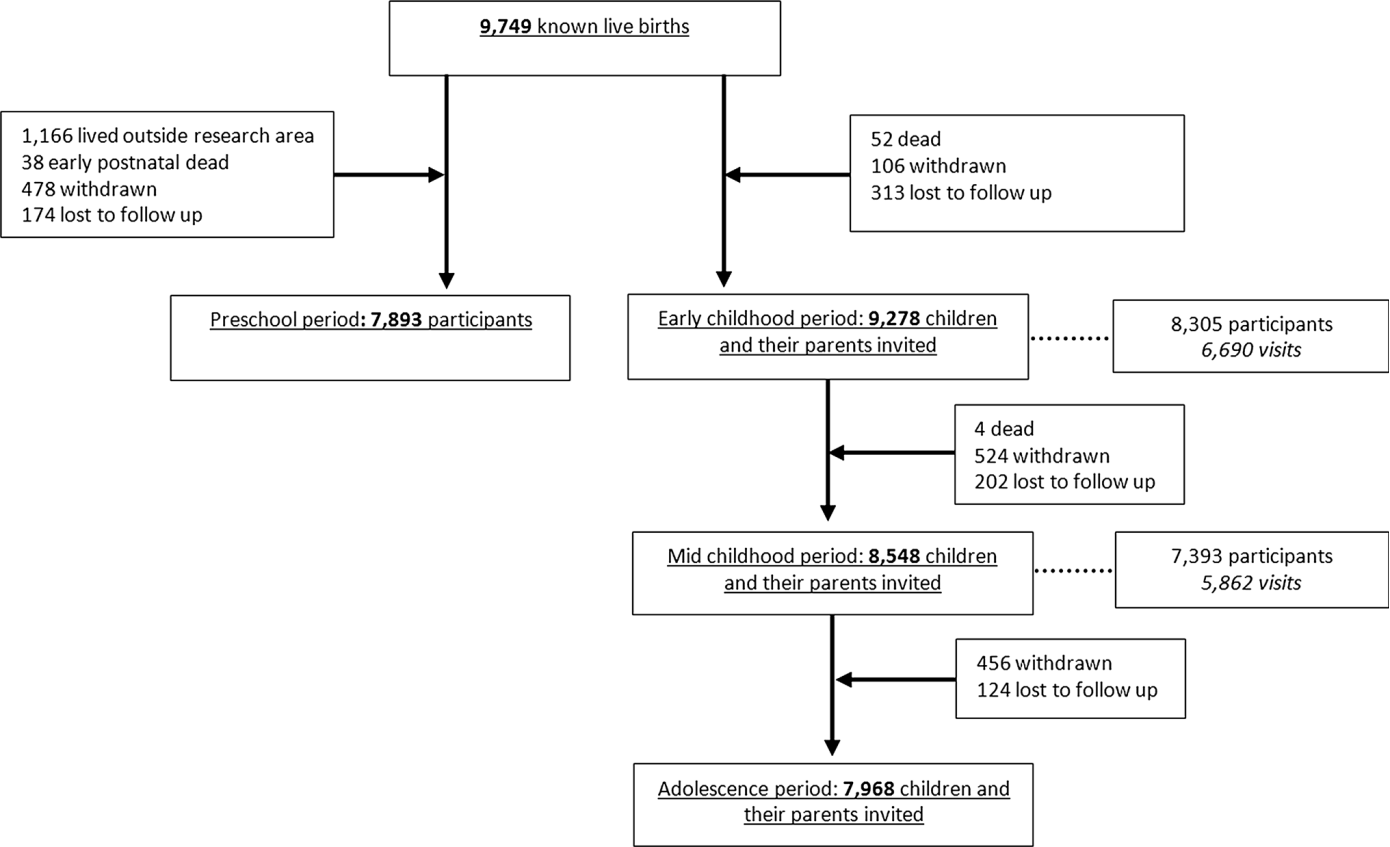
Enrolment and follow-up rates in the Generation R Study

Figure 1 shows the enrolment and follow-up rates of the children and parents included in the Generation R Study. The 9778 mothers enrolled in the study gave birth to 9749 live born children. During the preschool period (0–4 years), the logistics of the postnatal follow-up studies were embedded in the municipal routine child care system and restricted to only part of the study area. In total 1166 children lived outside this defined study area at birth and were therefore not approached for the postnatal follow-up studies during the preschool period. Of the remaining 8583 children, 690 (8%) parents did not give consent, or their children died or were lost to follow-up, leaving 7893 children for the preschool studies. At the age of 6 years (early school age), we invited all 9278 children from the original cohort of 9749 children to participate in follow-up studies. This invitation was independent of their home address and participation in the preschool period. In total, 8305 children (90% of those who were invited (n = 9278) and 85% of the original cohort (n = 9749)) still participated in the study at this age, of whom 6690 visited the research center at a median age of 6.0 years. For the follow-up phase at the age of 10 years (mid childhood period) 730 children of the 9278 could not be invited. In total, 7393 children (86% of those who were invited (n = 8548) and 76% of the original cohort (n = 9749)) participated in the study in mid childhood, of whom 5862 visited the research center at a median age of 9.7 years. Of the 8548 children invited in the mid childhood period, 456 had withdrawn and 124 children were lost to follow-up during this period, leaving 7968 children for invitation around the age of 13 (early adolescence period).

Table 3 shows the general characteristics of the mothers who were enrolled in the study at baseline, and who remaind in the study until the child’s age of 13 years. The median age of the women at enrolment was 30.5 (95% range, 19.3–39.6) years, 58% percent of those mothers were of the Dutch nationality, 43% of the mothers were highly educated and 55% had a high household income. The mean birth weight of the children was 3397 (SD 582) grams and they were born at a median gestational age of 40.0 (95% range, 34.9–42.3) weeks. Compared to the baseline characteristics, the mothers who still participated in the study at follow up were older, more frequently of Dutch nationality and higher educated.

**Table 3:** General characteristics

	Fetal period (n = 9749)	Preschool period 0–5 years (n = 7893)	Early school age/ Mid childhood period 6–11 years (n = 8305)	Adolescence period 12–16 years (n = 7968)
Mothers				
Age at enrolment (years)	30.5 (19.3, 39.6)	31.0 (19.6, 39.8)	31.1 (19.9, 39.9)	31.3 (20.0, 39.9)
Ethnicity				
Dutch, other-European (%)	58	61	64	65
Surinamese (%)	9	8	8	8
Moroccan (%)	6	6	6	5
Turkish (%)	8	8	8	7
Dutch Antilles (%)	3	2	2	2
Cape Verdian (%)	4	4	4	4
Others (%)	12	11	8	9
Educational level				
Low (no/primary education) (%)	11	10	9	8
Intermediate (secondary school, vocational training) (%)	46	43	42	41
High (Bachelor’s degree, University) (%)	43	47	49	51
Pre-pregnancy BMI	23.6 (4.4)	23.5 (4.2)	23.5 (4.1)	23.5 (4.1)
Net household income, per month				
<800 Euros (%)	9	8	7	6
800–2200 Euros (%)	36	34	32	32
>2200 Euros (%)	55	58	61	62
Children				
Sex				
Male (%)	51	51	51	50
Female (%)	49	49	49	50
Ethnicity				
Dutch, other-European (%)	62	65	67	68
Surinamese (%)	8	7	7	7
Moroccan (%)	7	6	6	6
Turkish (%)	8	8	7	6
Dutch Antilles (%)	4	3	3	3
Cape Verdian (%)	3	3	3	3
Others (%)	8	8	7	7
Birth weight (grams)	3397 (582)	3404 (572)	3412 (572)	3411 (576)
Gestational age at birth (weeks)	40.0 (34.9, 42.3)	40.0 (35.4, 42.3)	40.1 (35.4, 42.3)	40.1 (35.4, 42,3)

Values are means (standard deviation), percentages or medians (95% range)

## Measurements

### Data collection during pregnancy and fetal life

Physical examinations were planned at each visit in early pregnancy, mid pregnancy and late pregnancy and included height, weight and blood pressure measurements of both parents (Table 4).

**Table 4:** Assessments in mothers, fathers and their children during the fetal period

	Early pregnancy	Mid pregnancy	Late pregnancy	Birth
Mother				
Physical examination	+	+	+	
Questionnaire	+	+	+	
Interview			S	
Fetal growth ultrasound exam	+	+	+	
Fetal organ ultrasound exam			S	
Blood sample	+	+		
Urine sample	+	+	+	
Father (or partner)				
Physical examination	+	+^a^	+^a^	
Questionnaire		+		
Psychiatric interview			S	
Blood sample	+			
Child				
Physical examination				+
Cord blood				+

Early pregnancy: gestational age <18 weeks; mid pregnancy: gestational age 18–25 weeks; late pregnancy: gestational age >25 weeks

+ = Assessment in whole cohort

S = Assessment only in subgroup

^a^In case of intake at mid- or late pregnancy

Mothers received four postal questionnaires and fathers received one postal questionnaire during pregnancy. Topics in these questionnaires were:

- Mother 1: medical and family history, previous pregnancies, quality of life, life style habits, housing conditions, ethnicity, and educational level;
- Mother 2: diet, including macronutrients and micronutrients;
- Mother 3: current pregnancy, quality of life, life style habits, and psychopathology;
- Mother 4: current pregnancy, quality of life, life style habits, working conditions, household income, and self-esteem;
- Father: medical history, family history, life style habits, educational level, and psychopathology.

Blood samples were collected in early (mother, father) and mid-pregnancy (mother) and at birth (child). A detailed overview of the design and response of the biological sample collection and available measurements is given elsewhere [5, 7].

Fetal ultrasound examinations were performed at each prenatal visit. These ultrasound examinations were used to establish gestational age and to assess fetal growth patterns [35, 36]. These methods have previously been described in detail [37–39]. Longitudinal curves of all fetal growth measurements (head circumference, biparietal diameter, abdominal circumference and femur length) were created resulting in standard deviation scores for all of these specific growth measurements. Placental hemodynamics including resistance indices of the uterine and umbilical arteries have been measured in second and third trimester [40–42]. Detailed measurements of fetal brain, heart and kidney development were done in the subgroup [40, 43–48].

The obstetric records of mothers have been retrieved from hospitals and mid-wife practices to collect information about pregnancy progress and outcomes. Specialists in the relevant field coded items in these records [49].

### Data collection during the preschool period

At the age of 3 months, home visits were performed to assess neuromotor development using an adapted version of Touwen’s Neurodevelopmental examination and to perform a home environment assessment [50–53]. Information about growth (length (height), weight, head circumference) was collected at each visit to the routine child health centers in the study area using standardized procedures [54] (Table 5).

**Table 5:** Assessments in mothers, fathers and children during the preschool period

	Age (months)												
	2	3	4	6	11	12	14	18	24	30	36	45	48
Child													
Questionnaire (parent)	+	+		+		+		+	+	+	+		+
Physical examination	+	+	+	+	+		+		+		+	+	
Brain ultrasound	S												
Cardiac and renal ultrasound				S					S				
Blood pressure									S				
Airway inflammation				S					S				
Behavioural observation							S				S		S
Bacterial carriage	S			S			S		S		S		
Blood sample				S			S		S				
Mother													
Questionnaire		+		+							+		S
Interaction with child							S				S		
Father (or partner)													
Questionnaire											+		
Interaction with child													S

+ = Assessment in whole cohort

S = Assessment only in subgroup

**Fig. 2:**
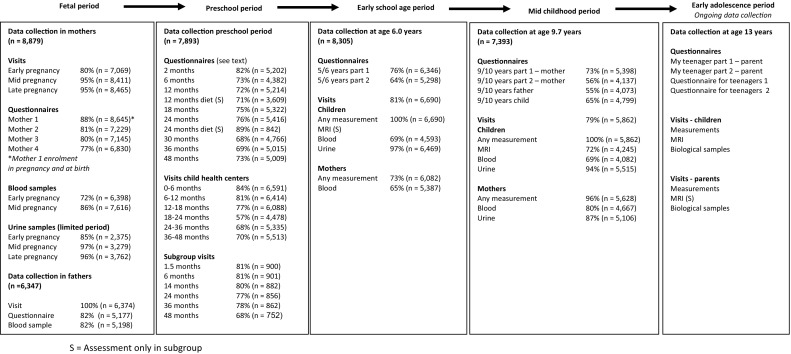
Response to the questionnaires and visits in the Generation R Study

During the preschool period, parents received 8 questionnaires, of which one was specifically for fathers. Items included in these questionnaires and their references are listed in Tables 6 and 7. Response rates based on the number of sent questionnaires are shown in Fig. 2. Not all children received each questionnaire due to logistical constraints and delayed implementation of some of the questionnaires after the first group of children reached the target age for those questionnaires. Thus, although response rates may be similar, the absolute number of completed questionnaires differs between different ages. Response rates presented in Fig. 2 are based on the number of sent questionnaires.

**Table 6:** Themes in postnatal questionnaires until early adolescence - Parental questionnaires

Main themes	2 months	6 months	12 months	18 months	24 months	30 months	36 months^e^	48 months	6 years^f^	10 years ^g^	13 years
Mother/father											
General health											
Quality of life [101]	+	+									
Pregnancy and complications	+								+		
Life events							+				
Medical history										+	
Lifestyle [102, 103]									+	+	+
Eating behaviour [104]											+
Social and demographic factors											
Housing and living conditions^a^ [105, 106]	+	+			+			+	+	+	+
Work and working conditions		+							+		+
Educational level and household income					+		+		+	+	+
Family activities and social support [107, 108]		+								+	
Mental health and stress											
Parenting [109, 110]				+			+		+		
Depressive symptoms [111]	+									+	
Psychopathology [112–114]	+	+					+			+	
Family functioning [115, 116]									+	+	
Child											
Diet and physical activity											
Diet^b^ [117, 118]	+	+	+		+/S				+		
Eating behaviour [119–127]					+			+	+	+	+
Television watching, use of computer and physical activity [128–131]					+		+	+	+	+	
Day-care, School		+	+				+		+	+	+
Childhood health and diseases											
Quality of life [132–135]					+		+		+	+	
Fever and infectious diseases [136]	+	+	+		+		+	+	+	+	
Asthma, Asthma related symptoms and eczema [137–140]		+	+		+		+	+	+	+	+
Acne [141]											+
Allergy		+	+							+	+
Accidents [142, 143]		+		+	+				+	+	+
Seizures^c^	+	+	+		+		+	+	+		
Abdominal pain, stool pattern [144]					+		+	+	+	+	
Doctors visit	+	+			+		+	+	+	+	
Teeth and dental care [145–148]									+		+
Physical characteristics									+		
Hearing (listen to music, use of headphone) [149]										+	+
Vision/Eyes (glasses, viewing habits (“close” and “far away”))										+	+
Behaviour and cognition											
Sleeping, crying and soothing [150–152]	+	+	+		+		+			+	
Temperament [153–156]	+								+		+
Motor development [157]		+	+	+	+			+			
Behaviour and emotional problems [158–161]		+		+			+		+	+	+
Pain perception [162–164]					+		+		+		
Language development [165]				+		+				+	+
Non-verbal cognition [166]					+	+					
Executive function [167]								+			
Prosocial behaviour [168–171]									+		+
Autistic traits [172–174]									+		+
Obsessive compulsive disorder [175]										+	+
Bullying									+		
Social media use [176, 177]											+

+ = Assessment in whole cohort. S = Assessment only in subgroup

^a^ Housing and living conditions include information about family structure, poverty, (environmental) smoking and pets

^b^ Diet questionnaires included in 2, 6 and 12 months questionnaire. Additional food frequency questionnaires at 12 months for all Dutch speaking children and at 24 months for the Dutch subgroup children

^c^ Screening 10 items questionnaire on seizures. Screen positives receive additional questionnaire and are being asked for their medical records

^d^ Infant Behaviour Questionnaire at the age of 6 months, Child Behaviour Checklist thereafter

^e^ For parenting, psychopathology and child behaviour additional questionnaire for fathers

^f^ Diet and part of behaviour and cognition additional at the age of 8 years

^g^ For medical history, lifestyle, depressive symptoms, psychopathology, family activities, behaviour and emotional problems additional questionnaire for fathers

**Table 7:** Themes in postnatal questionnaires—child questionnaire

Main themes	10 years	13 years
Friendships [161, 178]	+	+
Bullying [179–181]		+
General health [132]		+
Abdominal pain, stool pattern [182]		+
Social status [183]		+
Development and well-being [122, 184, 185]		+
Eating behaviour [126, 127, 186–189]	+	+
Television watching and physical activity [128, 131, 180, 181]	+	+
Temperament [182, 183]		+
Behaviour [161, 175, 194, 195]	+	+
Body Image [196, 197]	+	+
Self-perception [198–200]	+	+
Sleeping behaviour [201–204]	+	+
Puberty stages [203, 205]		+
Social media [176, 177]		+
Hearing (listen to music, use of headphone)		+
Vision (viewing habits (“close” and “far away”))		+

+ = Assessment in whole cohort

During the preschool period, children participating in the subgroup were invited six times to a dedicated research center. Measurements at these visits included physical examinations (height, weight, head circumference, skinfold thickness and waist—hip ratio, Touwen’s Neurodevelopmental Examination) and ultrasound examinations (brain, cardiac and kidney structures) [44, 55–59]. Dual X Energy Absorptiometry (DXA) scanning and Fractional exhaled Nitric Oxide (FeNO) measurements have been performed in a smaller subgroup [60, 61]. Blood pressure was measured at the age of 24 months [62, 63]. Observations of parent–child interaction and behaviour, such as executive function, heart rate variability, infant-parent attachment, moral development, and compliance with mother and child have been repeatedly performed and with father and child once [64–68]. Biological materials were collected if parents gave consent [69–71].

### Data collection during the early school age, mid childhood and adolescence period

From the age of 6 years onwards, we invite all participating children to a well-equipped and dedicated research center at the Erasmus MC-Sophia Children’s Hospital every 3–4 years. Visits at age 6 and 10 years have been completed, at age 13 years are ongoing and age 16 years are being planned.

Currently, the total visit takes about 3 h and all measurements are grouped in thematic 35 min blocks. Clinically relevant results are discussed with the children and their parents and, if needed, children or parents are referred to their general practitioner or other relevant health care provider.

At each age, we collect data using questionnaires on growth, health and physical and mental development of the children. Also, we collect information on childhood diet and behaviour (Table 6, 7). These questionnaires are sent to the primary caregiver.

The measurements at the research center are focused on several health parameters including behaviour and cognition, body composition, bone health and muscle function, eye development, growth, hearing, heart and vascular development, infectieus diseases and immunity, oral health and facial growth, respiratory health, allergy and skin disorders (Table 8) [72–79].

**Table 8:** Assessments in mothers and children during early school age, mid childhood and early adolescence visit

	Early school age (median age 6.0 (95% range 5.6–7.9) years)	Mid childhood (median age 9.7 (95% range 9.4–10.8) years)	Early adolescence (13 years, ongoing datacollection)
Mothers			
Behaviour and cognition			
Cognition	+		
Dutch language skills			+
Interaction with child		+	
Life events		+	
Interview about health, parenting, family situation, depression			+
Maternal health			
Anthropometrics and blood pressure	+	+	+
Arterial stiffness	+		
Endothelial function			+
Body composition and bone mineral density (DXA)	+	+	
Intima-media thickness		+	
Physical appearance	+	+	
Ultrasound heart	+		
Eyes; retinal vasculature, refraction	+		
Biological samples			
Blood sample		+	+
Urine sample		+	+
Hair sample	+		
Child			
Behaviour and cognition			
Behaviour and behavioural observation	+	+	+
Cognition	+	+	+
Language development	+	+	+
Pain perception	+		
Risk taking interview			+
Cardiovascular and metabolic development			
Anthropometrics and blood pressure	+	+	+
Arterial stiffness	+		
Body composition and bone mineral density (DXA)	+	+	+
Bone mineral density and geometry of the tibia (PQCT)		+	+
Intima-media thickness		+	+
Ultrasound abdominal fat	+		+
Ultrasound heart	+	+	
Ultrasound kidney	+		
Physical appearance		+	+
Puberty stages (Tanner)			+
Eyes, ears and mouth			
Eyes; visual acuity, retinal picture, refraction, IOL master, OCT	+	+	+
Dental status and development	+	+	+
Face development		+	+
Hearing		+	+
Taste experience	+		
Lungs			
Airway inflammation	+		
Lung function	+	+	+
Exercise test (SRT)			+
Allergy test		+	
Dermatology			
Spectrophotometry			+
Biological samples			
Nasopharynx bacterial carriage	+	+	
Blood and urine sample	+	+	+
Dental plaque			
Faeces microbiota		+	
Hair sample	+	+	+
Saliva	+	+	
Skin swab (head, elbow)			+

*DXA* Dual energy X-ray Absorptiometry scan, *PQCT* Peripheral quantitative computertomografie scan, *SRT* steep ramp test, *IOL* intraoculaire measurement, *OCT* optical coherence tomografie

S = assessment only in subgroup

We use various advanced imaging techniques including ultrasound and Doppler (GE LOGIQ E9, Milwaukee, WI, USA) for measuring thoracic and abdominal structures, Dual X Absorptiometry for measuring body composition and bone mineral density (iDXA scanner, GE Healthcare, Madison, WI, USA) and Peripheral Quantitative Computed Tomography (PQCT, Stratec Medicin Technik, Pforzheim, Germany) for measuring bone mineral density and geometry of the tibia. We use orthopantomograms (OP 200 D, Intrumentarium Dental, Tuusula, Finland) for measuring dental development.

MRI has been used for brain imaging in a subgroup (n = 801) of 6–8 year old children using a hospital-based 3.0 Tesla MRI scanner (Discovery MR750, GE Healthcare, Milwaukee, WI, USA) [80–83]. From 2014 onwards, we use a dedicated 3.0 Tesla MRI (Discovery MR750, GE Healthcare, Milwaukee, WI, USA) for brain and total body imaging of all children participating in the study at the mid childhood visit (age 10 years) (see Table 9 for the MRI outcome measures). We use a mock MRI scanner, to familiarize the children and get use to the scanning procedures. Children are scanned using standard imaging and positioning protocols, wearing light clothing without metal objects while undergoing the scanning procedure. Total scanning time amounts to approximately 60 min. The scanner is operated by trained research technicians and all imaging data are collected according to standardized imaging protocols. Changes or updates in hardware are avoided. Changes or updates in software configuration are minimized and regular checks with phantoms are performed to secure validity of cross-subject and cross-scan comparisons. Imaging is performed without administration of contrast agents. All imaging data are stored on a securely backed-up research picture archiving system, using programmed scripts to check for completeness of the data received. We will re-scanning the abdominal composition (fat), brain imaging and hip development during adolescence (age 13 years) of all participating children in Generation R. MRI scan of the brains will also be conducted in the parents of a subgroup of Generation R participants. This research is focused on aging effects of the brains in young adults and follow up of mothers who experienced gestational hypertensive complications.

**Table 9:** MRI measurements in children of the Generation R Study

	Early school age (median age 8.0 (95% range 6.3–10.1) years)	Mid childhood (median age 9.9 (95% range 9.5–11.9) years)	Early adolescence (13 years, ongoing datacollection)
Children			
Brain measurements			
Structural imaging			
3D T1-weighted GRE sequence	X(S)	X	X
2D-PD-weighted TSE sequence	X(S)	X	X
Diffusion tensor imaging (DTI)	X(S)	X	X
Resting state functional MRI	X(S)	X	X
Lungs			
Inspiratory volume		X	
Expiratory volume		X	
Sizes of the trachea		X	
Sizes of the main bronchi		X	
Chronic obstructive lung problems			
Air trapping		X	
Atelectasis		X	
Cardiac measurements			
Structural cardiac measurements		X	
Diastolic volume		X	
Cardiac mass		X	
Functional cardiac measurements		X	
Systolic volume		X	
Ejection fraction		X	
Stroke volume		X	
Aortic diameter		X (S)	
Total visceral adipose tissue from top of liver to femur head			
Fat volume/mass		X	x
Subcutaneous adipose tissue from top of liver to femur head			
Fat volume/mass		X	x
Pericardial fat			
Fat volume/mass		X	x
Kidney			
Length		X	
Width		X	
Depth		X	
Volume		X	
Liver			
Fat fraction		X	
Liver volume		X	
Structure and morphology of the hipbone		X	X
Testicular volume		X	
Ovarial volume		X	

S = assessment only in subgroup

Blood and urine samples are collected in the mothers and their children during every visit. A detailed overview of the design and response of the biological sample collection and available measures is given elsewhere [5, 7].

## Genomics: genetic, epigenetic and microbiome biobank

DNA from parents and children has been extracted and used for genotyping using taqman analyses for individual genetic variants and using a genome-wide association scan (GWAS) using the Illumina 670 K platform in the children [5, 7]. For genotyping, we used the infrastructure of the Human Genomics Facility (HuGe-F) of the Genetic Laboratory of the Department of Internal Medicine (www.glimdna.org). The GWAS dataset underwent a stringent QC process, which has been described in detail previously [5, 7, 84]. Most GWAS analyses are strongly embedded in the Early Growth Genetics (EGG) (http://egg-consortium.org/) and Early Genetics and Longitudinal Epidemiology (EAGLE) Consortia, in which several birth cohort studies combine their GWAS efforts focused on multiple outcomes in fetal life, childhood and adolescence. These efforts have already led to successful identification of various common genetic variants related to birth weight, infant head circumference, childhood body mass index, bone development and obesity and atopic dermatitis [85–91]. DNA from parents is used for genotyping for candidate gene or replication studies.

DNA methylation was measured on a genome wide level in a subgroup of Dutch children, using the Illumina Infinium HumanMethylation450 BeadChip (Illumina Inc., San Diego, USA). We used cord blood samples of 1339 children, blood samples in 469 children aged 6 years and blood samples in 425 children aged 10 years. Quality control and normalization of analyzed samples was performed using standardized criteria. Many of the epigenome-wide association analyses are performed in the context of the Pregnancy And Childhood Epigenetics (PACE) Consortium (http://www.niehs.nih.gov/research/atniehs/labs/epi/pi/genetics/pace/index.cfm), which brings together studies with epigenome-wide DNA-methylation data in pregnant women, newborns and/or children. Recent studies have identified differentially methylated sites in association with maternal smoking, maternal folate levels, maternal stress and air pollution during pregnancy [92–95].

Gut microbiota profiles were determined by Next Generation Sequencing (on Illumina MiSeq) of the V3 and V4 variable regions of the 16S ribosomal RNA gene in DNA extracted from feacal samples. Samples were collected at mid childhood in 2414 children. Phylogenetic *de novo* profiling was performed using the QIIME [96] and USEARCH [97] software packages and resulted in an operational taxonomic unit table with 239 species, 109 genera and 8 phyla. For example, those samples can be used for studying the effects of the fecel microbiota with overweight or obesity [98–100].

## Ethics

The general design, all research aims and the specific measurements in the Generation R Study have been approved by the Medical Ethical Committee of Erasmus MC, University Medical Center Rotterdam. New measurements are only introduced into the study after approval of the Medical Ethical Committee. Participants need to give written informed consent for each phase of the study (fetal, preschool, childhood and adolescence period). From the age of 12 years onwards, children must sign their own consent form, in accordance with Dutch Law. At the start of each phase, children and their parents receive written and oral information about the study. Even with consent, when the child or the parents are not willing to participate actively, specific measurements are skipped or no measurements at all are performed.

## Follow-up and retention strategies

Thus far, loss to follow-up has been lower than 10%. Major efforts are made to keep the children and parents involved in the study and to minimize loss to follow-up. Several strategies have been implemented and are currently part of the study design:

- Addresses: new addresses of participants, which are known by the municipal health service, can be retrieved by the study staff;
- Newsletters: participants receive two to four newsletters per year, in which several results of the study are presented and explained, questions of participants are answered and new research initiatives are presented;
- Facebook: every week we post a short news update about the ongoing research on our facebook page;
- Website: we have an up-to-date website where participants can find information about the ongoing research, the procedures at the dedicated research center and our contact information;
- Presents and discounts: all children who visit our research center receive small presents. Also, discount offers are regularly presented in the newsletter;
- Transport costs: all costs for transport and parking related to visits to the research center are reimbursed;
- Reminders for questionnaires: when the questionnaire has not been returned within 3 weeks, a kind reminder letter is sent to the parents. After 6 weeks, if the questionnaire still has not been returned, the parents receive a phone call. If necessary, help with completing the questionnaire is offered and the importance of filling out the questionnaire is explained once more during this phone call;
- Individual feedback: if clinically relevant, results of measurements are discussed with the parents and children at the visit. If necessary, follow-up appointments with the general practitioner are planned;
- Support for non-Dutch speaking participants: all study materials such as questionnaires, newsletters, website, and information folders are available in three languages (Dutch, English, and Turkish). Furthermore, staff from different ethnic backgrounds is available and verbally translate these materials into Arabic, French and Portuguese. As such, the study staff is able to communicate with all participants;
- Additional help: children and parents who showed low response rates for different measurements, showed difficulties in completing questionnaires or require additional explanation or support are pro-actively contacted by one dedicated member of the study staff;
- Home visits: We visit children and parents who cannot be contacted by phone, e-mail or letter. Most visits are planned in the evenings to have higher chances that both parents and children are at home.

## Power, datamanagement, privacy protection

Power calculations for the Generation R Study are shown in Tables 10 and 11. Due to missing values and loss to follow-up, most analyses in the study are not based on data in all subjects. Therefore, these power calculations demonstrated are based on 7000 subjects in the whole cohort and 700 subjects in the subgroup. The presented power calculations are conservative since most studies will assess the effects of continuous instead of dichotomous exposures and studies may be focused on outcomes collected in more than only 1 year.

**Table 10:** Effects sizes that can minimally be detected according to the prevalence of the exposure

Proportion exposed (%)	Whole cohort (n = 7000)	Subgroup (n = 700)
50	0.067	0.212
25	0.077	0.276
10	0.112	0.353
5	0.154	0.486
1	0.337	1.064

The presented effect sizes are detectable proportions of the standard deviation with a type I error of 5% and a type II error of 20% (power 80%)

**Table 11:** Relative risks that can minimally be detected according to the prevalence of the exposure

Proportion exposed (%)	Incidence (1 year) of outcome of interest					
	Whole cohort (n = 7000)			Subgroup (n = 700)		
	10%	5%	1%	10%	5%	1%
50	1.23	1.33	1.83	1.83	2.28	4.94
25	1.26	1.38	1.94	1.96	2.46	5.41
10	1.39	1.56	2.42	2.48	3.26	7.92
5	1.55	1.80	3.09	3.20	4.39	11.74
1	2.36	3.04	6.83	7.75	11.61	37.55

The presented effect sizes are detectable relative risks with a type I error of 5% and a type II error of 20% (power 80%)

From 2016 onwards, data collected during the measurements at the research center are entered directly into an electronic database. Data collected by questionnaires are scanned and manually entered into an electronic database by a commercial company. Random samples of all questionnaires are double checked by study staff members to monitor the quality of this manual data entry process. The percentage of mistakes does not exceed 3% per questionnaire. Open text fields are entered into the electronic database exactly as they are filled in on the questionnaires. In a secondary stage, these open text fields are cleaned and coded by a specialist in the relevant field.

All measurements are centrally checked by examination of the data including their ranges, distributions, means, standard deviations, outliers and logical errors. Data outliers and missing values are checked with the original forms. The data of one specific measurement are only distributed for analyses after data collection and preparation is completed for that measurement for the whole cohort.

Datasets needed for answering specific research questions are centrally constructed from different databases. All information in these datasets that enables identification of a particular participant, including names and dates of birth, is excluded before distribution to the researchers. The datasets for researchers include unique identification numbers for each subject that enable feedback about individuals to the datamanager but do not enable identification of that particular subject. Currently, we are exploring possibilities for a remote access environment, in which researchers can access centrally stored research data from their own computer without storing such data locally.

## Collaboration

The Generation R Study is conducted by several research groups from the Erasmus MC in close collaboration with the Erasmus University Rotterdam and the Municipal Health Service Rotterdam area. Since the data collection is still ongoing and growing, the number of collaborating research groups in and outside the Netherlands is expected to increase. Various research projects are performed as part of ongoing European or worldwide collaboration projects. The study has an open policy with regard to collaboration with other research groups. Request for collaboration can be sent to Vincent Jaddoe (v.jaddoe@erasmusmc.nl). These requests will be discussed in the Generation R Study Management Team regarding their study aims, overlap with ongoing studies, logistic consequences and related finances. After approval of a project by the Generation R Study Management Team and the Medical Ethical Committee of Erasmus MC, the collaborative research project is embedded in one of the research areas supervised by the corresponding principal investigator.
